# IMPACT OF OFFLINE AND ONLINE SOCIAL NETWORKS ON LONELINESS AND MENTAL HEALTH AMONG ITALIAN OLDER PEOPLE

**DOI:** 10.1093/geroni/igz038.1945

**Published:** 2019-11-08

**Authors:** Daniele Zaccaria, Georgia Casanova, Antonio Guaita

**Affiliations:** Golgi Cenci Foundation, Abbiategrasso, Italy

## Abstract

In the last decades the study of older people and social networks has been at the core of gerontology research. The literature underlines the positive health effects of traditional and online social connections and also the social networks’s positive impact on cognitive performance, mental health and quality of life. Aging in a Networked Society is a randomized controlled study aimed at investigating causal impact of traditional face-to-face social networks and online social networks (e.g. Social Network Sites) on older people’ health, cognitive functions and well-being. A social experiment, based on a pre-existing longitudinal study (InveCe - Brain Aging in Abbiategrasso) has involved 180 older people born from 1935 to 1939 living in Abbiategrasso, a municipality near Milan. We analyse effects on health and well-being of smartphones and Facebook use (compared to engagement in a more traditional face-to-face activity), exploiting the research potential of past waves of InveCe study, which collected information concerning physical, cognitive and mental health using international validate scale, blood samples, genetic markers and information on social networks and socio-demographic characteristics of all participants. Results of statistical analysis show that poor social relations and high level of perceived loneliness (measured by Lubben Scale and UCLA Loneliness scale) affect negatively physical and mental outcomes. We also found that gender and marital status mediate the relationship between loneliness and mental wellbeing, while education has not significant effect. Moreover, trial results underline the causal impact of ICT use (smartphones, internet, social network sites) on self-perceived loneliness and cognitive and physical health.

